# The relationship between the C-reactive protein-triglyceride glucose index and gallstones in American adults: Results from NHANES 2017 to 2020

**DOI:** 10.1097/MD.0000000000044650

**Published:** 2025-09-19

**Authors:** Qiu Su, Saimei Yang, Qiyi Liu

**Affiliations:** a Department of General Surgery, Beiliu City People’s Hospital, Yulin, China.

**Keywords:** C-reactive protein triglyceride-glucose index, CTI, gallstones, inflammation, insulin resistance, NHANES

## Abstract

Research has revealed that the C-reactive protein-triglyceride glucose index (CTI) reliably indicates the presence of inflammation and insulin resistance. These are both key factors in causing gallstones. This cross-sectional study investigated the potential link between CTI and the development of gallstones, analyzing data from the 2017 to 2020 National Health and Nutrition Examination Survey. CTI was calculated using the following formula: 0.412 × Ln (C-reactive protein) (mg/L) + Ln [triglycerides (mg/dL) × fasting blood glucose (mg/dL)/2]. Gallstones were identified through self-reports. Logistic regression analysis was used to assess the relationship between CTI and gallstones. Subgroup analyses, interaction tests and smoothed curve fitting were also conducted to ensure the robustness of the findings. The study included a total of 3861 participants. The results revealed a significant association between CTI and gallstones (odds ratio: 1.23, 95% confidence interval: 1.06–1.42, *P* = .005). Those in the highest CTI quartile were more likely to have gallstones than those in the lowest quartile (odds ratio: 2.02, 95% confidence interval: 1.38–2.96, *P* < .001). Consistently, both the smoothed curve fitting and the subgroup analysis highlighted a positive correlation. Indicating that an elevated CTI level could be a valuable clinical indicator for identifying gallstones.

## 1. Introduction

Gallstone disease is a major global health concern. This is because it is very prevalent and causes complications. It is characterized by the formation of cholesterol or pigment stones in the gallbladder or bile ducts. It is estimated that 10% to 20% of adults worldwide have gallstones, with a higher prevalence in Western populations, especially women and individuals with metabolic diseases such as obesity and type 2 diabetes.^[1,2]^

In the United States alone, gallstones result in over 1.8 million hospitalizations each year, costing the healthcare system around $6.5 billion.^[3]^ Complications such as acute cholecystitis, pancreatitis, and cholangitis result in a severe disease burden, and the increasing prevalence of metabolic syndrome portends a growing burden of this disease in the coming decades.^[4]^ Despite advances in laparoscopic cholecystectomy, the economic and social impact of gallstone disease highlights the urgent need for early gallstone risk stratification.^[5,6]^ Gallstone formation results from the interaction of multiple factors, including genetic susceptibility, oversaturation of cholesterol in bile and diminished gallbladder contractility.^[7]^ Recent studies have identified inflammation and insulin resistance as factors that regulate these pathways. Chronic inflammation, as indicated by elevated C-reactive protein (CRP) levels, promotes cholesterol precipitation by altering hepatic lipid metabolism and bile acid synthesis.^[8]^ At the same time, insulin resistance exacerbates biliary cholesterol overproduction by dysregulating sterol regulatory element-binding proteins, leading to cholecystic stagnation through autonomic neuropathy.^[2]^ Insulin resistance is indicated by the triglyceride-glucose index (TyG). It is worked out from a blood test. It can show problems with the body’s metabolism. Elevated TyG levels have been shown to be a risk factor for gallstone disease in previous research.^[9]^ The C-reactive protein-triglyceride-glucose index (CTI) reflects inflammation and insulin resistance. First proposed in 2022, it calculates a score based on CRP and TyG values, and has been shown to have prognostic value in cancer and cardiovascular disease (CVD).^[10–13]^ Furthermore, the new CTI is a cost-effective, accessible and rapid biochemical screening index. Previous studies have primarily examined the relationship between individual biomarkers, such as CRP or the TyG index, and gallstones,^[14–16]^ This restricts the study’s reliability and generalizability. However, the potential link between CTI and gallstones remains to be explored. Taking these limitations into account, the objective of this study is to comprehensively examine the association between CTI and gallstones. The publicly available U.S. National Health and Nutrition Examination Survey (NHANES) population dataset provided a solid foundation for our research. The hypothesis was made that a significant positive correlation between CTI and gallstones would be found, with this correlation remaining consistent at higher CTI quartiles. Our study could improve the way gallstones are assessed and help to prevent them through a combination of different interventions.

## 2. Methods

### 2.1. Study design and population

**Figure 1.**
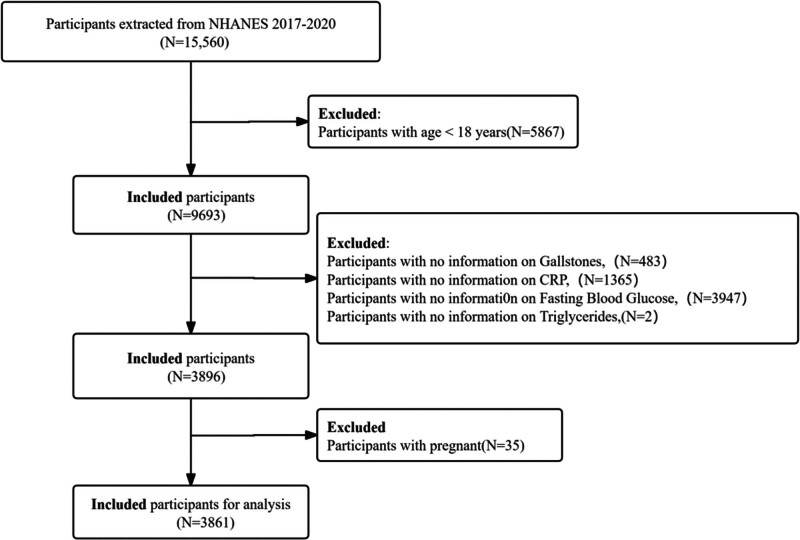
Flowchart showing the selection process of the study population.

This study looked at information from the NHANES, which was done between 2017 and 2020. The study adhered to STROBE criteria of observational studies. It was carried out by the U.S. Centers for Disease Control and Prevention. The NHANES project tries to find out about the health and diet of American people who do not live in hospitals or other places that look after people. The people who take part in the study are chosen in a special way. The initiative encompasses home visits, screening procedures and laboratory tests conducted by mobile examination centers to collate demographic and exhaustive health information. The NHANES study was approved by the Ethics Review Board of the National Center for Health Statistics, and written informed consent was provided by all participants prior to their involvement. Secondary analysis of the data did not require additional institutional review board approval. NHANES data can be accessed via the NHANES website (http://www.cdc.gov/nchs/nhanes.htm). For this study, publicly available data from 2017 to 2020 were incorporated. Participants extracted from NHANES 2017 to 2020 (N = 15,560). Participants were excluded if their data met any of the below criteria: participants aged under 18 years; missing data relating to CTI indicators (including triglycerides, fasting glucose, and CRP); missing data relating to gallstone disease; and pregnant participants. After applying these criteria, 3861 individuals were selected from the initial group of 15,560 participants (see Fig. 1).

### 2.2. Assessment of CTI

In line with previous studies,^[12,17]^ CTI was determined using biochemical data obtained from blood samples, including CRP, triglycerides, and fasting glucose. Triglyceride and fasting glucose levels serve as markers of insulin resistance. Blood samples were collected in the morning after an overnight fast of at least 8 hours, then transported to a laboratory certified by the National Center for Health Statistics. Measurements of CRP, triglycerides, and fasting glucose were conducted using the Latex Enhanced Turbidimetric Method on a Behring nephelometer, the Enzymatic Method on a Roche Cobas 6000 Chemistry Analyzer (Hitachi High-Technologies Corporation, Tokyo, Japan) and the Oxygen Rate Method on a Beckman DxC800 (Beckman Coulter, Inc., Brea), respectively. CTI was calculated using the following formula: CTI = 0.412 × ln(CRP) (mg/L) + ln[triglycerides (mg/dL) × fasting blood glucose (mg/dL)/2].^[10]^

### 2.3. Gallstone assessment

Participants were diagnosed with gallstone disease based on their responses to the following question: “Has a doctor or other healthcare provider ever told you that you have gallstones?.”^[18,19]^

### 2.4. Assessment of potential covariates

For our study, 4 categories of variables were selected as covariates: sociodemographic characteristics, behavioral traits, comorbid conditions and laboratory test results.^[11,17,20]^ *Sociodemographic characteristics*: Age was divided into 2 groups: under 60 years and 60 years and over. Gender was classified as female or male. Ethnicity was categorized as non-Hispanic white, non-Hispanic black, Mexican American, other Hispanic or other ethnicities. The poverty-income ratio was categorized as <1.3, between 1.3 and 3.5, or 3.5 or higher. Education level was categorized as <9 years, 9 to 12 years, and 12 years or more. Marital status was defined as living alone or being married/living with a partner. *Behavioral traits*: These included body mass index (BMI), smoking status and alcohol consumption. BMI was classified as under 25, 25 to 30, or 30 or higher. It was calculated by dividing weight in kilograms by height in meters squared (kg/m²). Smoking status was categorized as never smoker (fewer than 100 cigarettes in lifetime), former smoker (>100 cigarettes in lifetime but not currently smoking), or current smoker (>100 cigarettes in lifetime and currently smoking).^[21]^ Alcohol use was divided into no alcohol use (fewer than 12 drinks in the past year) and alcohol use (12 or more drinks in the past year). *Comorbid conditions*: These included hypertension, diabetes mellitus (DM), hyperlipidemia, CVD, and chronic kidney disease (CKD). Hypertension and DM were identified through self-reports. Hyperlipidemia was determined by a total cholesterol level of 200 mg/dL or higher, or by the use of lipid-lowering medication.^[22,23]^ CVD was identified through self-reported conditions such as congestive heart failure, coronary artery disease, or heart attack. CKD was defined as an estimated glomerular filtration rate (eGFR) of <60 mL/min/1.73 m², as calculated using the Chronic Kidney Disease Epidemiology Collaboration equation.^[17,24]^ *Laboratory test results*: These included uric acid, total cholesterol, high-density lipoprotein, glycated hemoglobin, creatinine concentration, and eGFR. In NHANES, biochemical measurements were conducted during the same investigative cycle, with intervals between measurements ranging up to 7 days.

### 2.5. Statistical analysis

This study is a secondary analysis of a publicly accessible dataset. Missing covariate data were imputed using multiple interpolation techniques. Five final datasets were generated and integrated in total. Categorical variables are presented as proportions (%), while continuous variables are characterized by the mean (standard deviation) or the median (interquartile range), as appropriate. One-way ANOVA was employed to compare differences between groups with normally distributed data, the Kruskal–Wallis test was used for skewed distributions and the Chi-squared test was used for categorical variables. Logistic regression models were used to estimate the odds ratio (OR) and 95% confidence intervals (CIs) for the association between CTI and gallstones. Model 1: no covariates were adjusted. Model 2 accounted for sociodemographic factors including age, gender, race/ethnicity, marital status, educational level, and household income. Model 3 included the aforementioned adjustments, as well as smoking status, alcohol use, hypertension, DM, hyperlipidemia, CKD, CVD, uric acid, hemoglobin A1c, creatinine, and eGFR. Restricted cubic spline regressions were conducted with 4 nodes positioned at the 5th, 35th, 65th, and 95th percentiles of CTI, in order to evaluate linearity and explore the dose–response relationship between CTI and gallstones. This was done after adjusting for the covariates in Model 3. Furthermore, potential modifications to the relationship between CTI and gallstones were examined across various variables: gender; age (18–60 years vs >60 years); marital status (married or cohabiting vs living alone); educational attainment (<9 years vs 9–12 years or >12 years); and household income (low vs medium or high). Heterogeneity among subgroups was evaluated using multivariate logistic regression, and interactions between subgroups and CTI were tested using likelihood ratio tests. All statistical analyses were conducted using R Statistical Software (version 4.2.2; The R Project for Statistical Computing; The R Foundation, Vienna, Austria) and the FreeStatistics analysis platform (version 2.1.1; FreeStatistics, Beijing, China). FreeStatistics provides user-friendly interfaces for common analyses and data visualization, utilizing R as its core statistical engine and employing a Python-based graphical user interface. Most analyses can be performed with minimal user input, thereby promoting reproducibility and interactive computing. A two-sided *P*-value of <.05 was considered statistically significant.^[25,26]^

## 3. Results

### 3.1. Characteristics of study participants

Table 1 summarizes the detailed intergroup differences in clinical characteristics. A total of 3861 participants were included in the study, of whom 418 were diagnosed with gallstones and 3443 were not. The study found that individuals with gallstones were older (mean age: 58.3 ± 15.4 years vs 50.3 ± 17.3 years, *P* < .001), had a higher proportion of females (71.8% vs 28.2%, *P* < .001) and had a higher BMI (mean BMI: 33.3 ± 8.7 vs 29.6 ± 7.3, *P* < .001). There were also significant differences in metabolic markers, with those with gallstones presenting higher fasting glucose levels (121.1 ± 42.0 vs 112.9 ± 37.3 mg/dL), a higher tyG index (8.7 ± 0.7 vs 8.5 ± 0.7), and higher CRP levels (2.8 vs 1.9 mg/dL) (all *P* < .001). The prevalence of comorbidities also differed, with hypertension (48.3% vs 30.0%), DM (29.2% vs 15.3%), and CVD (18.7% vs 8.6%) being more prevalent in patients with gallstones (all *P* < .001). However, no significant differences were observed in uric acid, creatinine, or total cholesterol levels between the 2 groups (*P* > .05).

**Table 1:** Baseline characteristics of study participants based on gallstones from NHANES 2017 to 2020.

Characteristics	Total participants	Gallstones		*P*-value
		NO	YES	
Number, n	3861	3443	418	
Age, yr	51.1 ± 17.3	50.3 ± 17.3	58.3 ± 15.4	<.001
*Age, strata (%*)				<.001
<60	2443 (63.3)	2241 (65.1)	202 (48.3)	
≥60	1418 (36.7)	1202 (34.9)	216 (51.7)	
*Sex, strata (%*)				<.001
Male	1882 (48.7)	1764 (51.2)	118 (28.2)	
Female	1979 (51.3)	1679 (48.8)	300 (71.8)	
*Race, %*				<.001
Mexican American	496 (12.8)	437 (12.7)	59 (14.1)	
Non-Hispanic White	399 (10.3)	344 (10)	55 (13.2)	
Non-Hispanic Black	1304 (33.8)	1136 (33)	168 (40.2)	
Other Hispanic	970 (25.1)	899 (26.1)	71 (17)	
Other races	692 (17.9)	627 (18.2)	65 (15.6)	
PIR, continuous	2.6 ± 1.6	2.6 ± 1.6	2.5 ± 1.5	.263
*PIR, strata (%*)				.04
<1.3	897 (27.0)	805 (27.2)	92 (25.1)	
1.3–3.5	1345 (40.4)	1175 (39.7)	170 (46.4)	
>3.5	1086 (32.6)	982 (33.2)	104 (28.4)	
*Educational level, %*				.562
<9	310 (8.0)	279 (8.1)	31 (7.4)	
9–12	1361 (35.3)	1204 (35)	157 (37.6)	
>12	2188 (56.7)	1958 (56.9)	230 (55)	
*Marital status, %*				.699
Married or living with a partner	2260 (58.5)	2019 (58.6)	241 (57.7)	
Living alone	1601 (41.5)	1424 (41.4)	177 (42.3)	
BMI, continuous,kg/m^2^	29.9 ± 7.6	29.6 ± 7.3	33.3 ± 8.7	<.001
*BMI, strata (%*)				<.001
<25	980 (25.8)	924 (27.3)	56 (13.8)	
25–30	1219 (32.1)	1112 (32.8)	107 (26.3)	
>30	1598 (42.1)	1354 (39.9)	244 (60)	
*Smoking status, %*				.004
Never	2195 (56.9)	1967 (57.1)	228 (54.8)	
Former	944 (24.5)	817 (23.7)	127 (30.5)	
Current	719 (18.6)	658 (19.1)	61 (14.7)	
*Alcohol use, %*				.36
YES	34 (1.3)	33 (1.4)	1 (0.4)	
NO	2524 (98.7)	2297 (98.6)	227 (99.6)	
HbA1c	5.9 ± 1.2	5.9 ± 1.1	6.1 ± 1.3	<.001
Uric acid, mg/dL	5.5 ± 1.5	5.5 ± 1.5	5.5 ± 1.5	.45
Creatinine, mg/dL	0.9 ± 0.5	0.9 ± 0.5	0.9 ± 0.6	.69
Total cholesterol, mg/dL	183.9 ± 41.0	184.1 ± 40.6	181.9 ± 43.8	.294
High-density lipoprotein, mg/dL	53.7 ± 16.0	53.8 ± 16.2	52.3 ± 14.0	.056
eGFR	100.0 ± 30.7	100.4 ± 30.1	96.8 ± 34.7	.023
Fasting glucose, mg/dL	113.7 ± 37.9	112.9 ± 37.3	121.1 ± 42.0	<.001
Fasting triglyceride, mg/dL	89.0 (60.0, 132.0)	88.0 (59.0, 131.0)	105.5 (71.2, 146.0)	<.001
TyG	8.5 ± 0.7	8.5 ± 0.7	8.7 ± 0.7	<.001
CRP, mg/L	2.0 (0.8, 4.4)	1.9 (0.8, 4.2)	2.8 (1.2, 5.7)	<.001
CTI	8.8 ± 0.9	8.8 ± 0.9	9.1 ± 0.9	<.001
*Hypertension, %*				<.001
YES	1232 (32.0)	1030 (30)	202 (48.3)	
NO	2618 (68.0)	2402 (70)	216 (51.7)	
*Diabetes mellitus, %*				<.001
YES	629 (16.8)	510 (15.3)	119 (29.2)	
NO	3114 (83.2)	2826 (84.7)	288 (70.8)	
*Hyperlipidemia, %*				.003
YES	1402 (45.4)	1202 (44.4)	200 (52.6)	
NO	1683 (54.6)	1503 (55.6)	180 (47.4)	
*CVD, %*				<.001
YES	374 (9.7)	296 (8.6)	78 (18.7)	
NO	3487 (90.3)	3147 (91.4)	340 (81.3)	
*CKD, %*				.085
NO	2248 (58.3)	1988 (57.8)	260 (62.2)	
YES	1609 (41.7)	1451 (42.2)	158 (37.8)	

BMI = body mass index; CKD = chronic kidney disease; CRP = C-reactive protein; CTI = C-reactive protein-triglyceride glucose index; CVD = cardiovascular disease; eGFR = estimated glomerular filtration rate; HbA1c = hemoglobin A1c; NHANES = National Health and Nutrition Examination Survey; PIR = poverty income ratio; TyG = triglyceride-glucose index.

### 3.2. Association between CTI and gallstones

**Figure 2.**
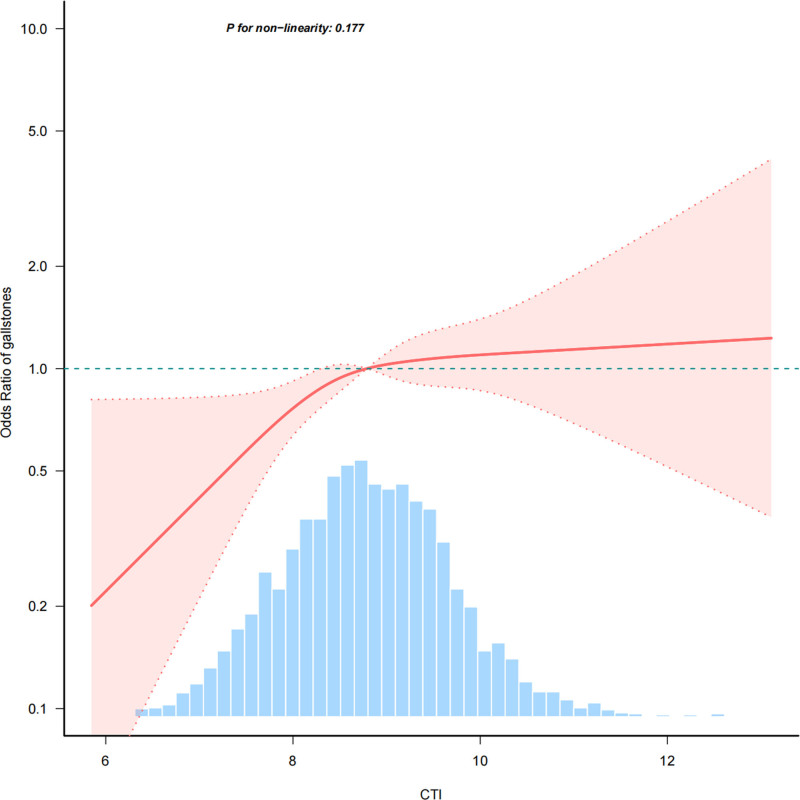
Association between CTI and gallstones odds ratio. Solid and dashed lines. Represent the predicted value and 95% confidence intervals. They were adjusted for age, sex, race, educational level, and marital status, PIR, smoking status, alcohol use, HbA1c, creatinine, uric acid, eGFR, hypertension, diabetes mellitus, hyperlipidemia, CVD, and CKD were adjusted. CKD = chronic kidney disease, eGFR = estimated glomerular filtration rate, PIR = poverty income ratio.

In our analysis of the relationship between CTI and gallstones, we adopted a quartile-based approach to assess the risk associated with different levels of CTI. The results, presented in Table 2, demonstrate a significant positive correlation between CTI and gallstone prevalence. Model 1 showed that the OR increased by 1.47 for every one-unit increase in CTI (95% CI: 1.32–1.63; *P* < .001). This decreased to 1.34 (95% CI: 1.19–1.51; *P* < .001) after adjusting for Model 2 and to 1.23 (95% CI: 1.06–1.42; *P* = .005) after adjusting for Model 3. When the data were stratified by quartile, the highest quartile (CTI ≥ 9.42) exhibited a notably higher gallstone risk, with an OR of 3.32 (95% CI: 2.38–4.62; *P* < .001) in Model 1. This remained significant after adjusting for Model 3 (OR = 2.02, 95% CI: 1.38–2.96; *P* < .001). A significant trend was observed across quartiles in all models (*P* for trend < .001 in Models 1 and 2 and *P* = .003 in Model 3), indicating a dose–response relationship. Furthermore, this study employed smoothed curve analysis to investigate the correlation between CTI and gallstones. As shown in Figure 2, curve fitting indicated a positive linear relationship between increased CTI levels and gallstone prevalence.

**Table 2:** Associations between CTI and gallstones.

Quartiles	OR (95% CI)						
	No.	Model 1	*P* value	Model 2	*P* value	Model 3	*P* value
CTI (per 1 unit)	3861	1.47 (1.32–1.63)	<.001	1.34 (1.19–1.51)	<.001	1.23 (1.06–1.42)	.005
Q1 (≤8.17)	965	1 (Ref)		1 (Ref)		1 (Ref)	
Q2 (8.17–8.79)	965	2.23 (1.58–3.16)	<.001	1.88 (1.31–2.68)	.001	1.84 (1.28–2.64)	.001
Q3 (8.79–9.42)	965	2.28 (1.62–3.22)	<.001	1.8 (1.26–2.57)	.001	1.63 (1.13–2.36)	.009
Q4 (≥9.42)	966	3.32 (2.38–4.62)	<.001	2.53 (1.79–3.58)	<.001	2.02 (1.38–2.96)	<.001
Trend.test	3861		<.001		<.001		.003

Model 1: no covariates were adjusted.

Model 2: age, sex, race, educational level, and marital status, and PIR were adjusted.

Model 3: age, sex, race, educational level, and marital status, PIR, smoking status, alcohol use, HbA1c, creatinine, uric acid, eGFR, hypertension, diabetes mellitus, hyperlipidemia, CVD, and CKD were adjusted.

95% CI = 95% confidence interval; CKD = chronic kidney disease; CTI = C-reactive protein-triglyceride glucose index; CVD = cardiovascular disease; eGFR = estimated glomerular filtration rate; HbA1c = hemoglobin A1c; OR = odds ratio; PIR = poverty income ratio.

### 3.3. Subgroup analyses

**Figure 3.**
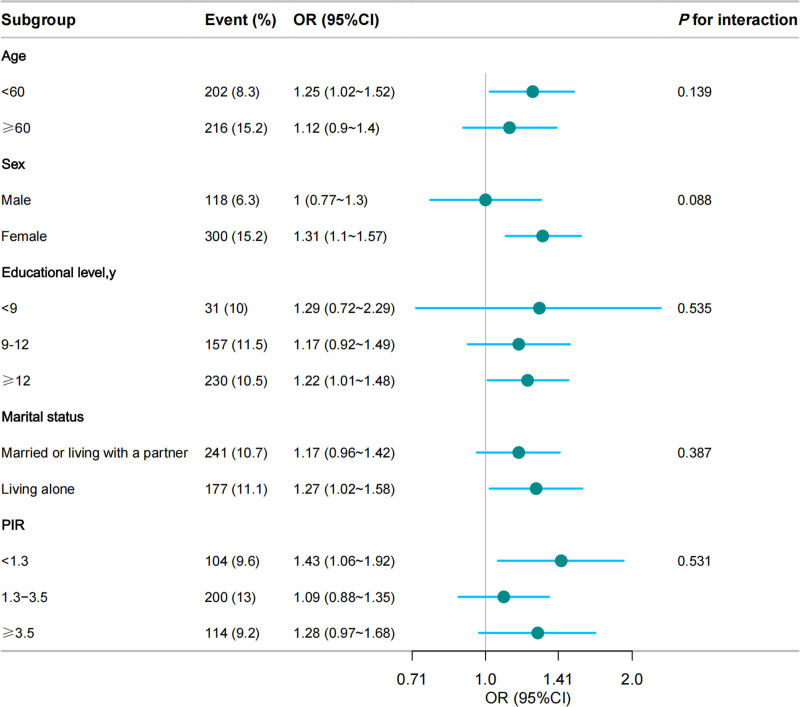
The relationship between CTI and gallstones according to basic features. Except for the stratification component itself, each stratification factor was adjusted for all. Other variables (age, sex, race, educational level, and marital status, PIR, smoking status, alcohol use, HbA1c, creatinine, uric acid, eGFR, hypertension, diabetes mellitus, hyperlipidemia, CVD, and CKD). CKD = chronic kidney disease, CVD = cardiovascular disease, eGFR = estimated glomerular filtration rate, PIR = poverty income ratio.

To evaluate the consistency and reliability of the relationship between CTI and gallstones in Model 3 following full adjustment, subgroup analyses and interaction tests are conducted. A positively correlated relationship between CTI and gallstones was consistently observed across various stratification factors, comprising gender, age, marital status, educational status, and household income. As Figure 3 (the forest plot) shows, nonsignificant *P*-values (*P* > .05) were obtained for all subgroups in the interaction tests, indicating that the impact of the stratification factors on the relationship was uniform.

## 4. Discussion

Using NHANES data, we performed cross-sectional analyses and established a positive correlation between CTI and gallstone risk. It was shown by a fully adjusted logistic regression model that all covariates were considered that individuals in the higher CTI quartiles were more likely to have gallstones. Smoothed curve analysis revealed a consistent linear trend, and subgroup analyses using different stratification factors confirmed the link between CTI and gallstones.

In our study, we found that older people and women were at a higher risk of developing gallstones, a fact that has been confirmed in several studies. For example, a case-control study of patients with gallbladder cancer identified 5 independent risk factors for gallstones (including female patients and those of advanced age).^[27]^ Additionally, research using information from the NHANES found a big increase in the number of people getting gallstones as they get older (the average age of people with gallstones vs those without was 56.435 vs 46.896 years, *P* < .001), and a higher rate among women (75.1% vs 24.9%, *P* < .001).^[28]^ Our study’s findings align with these results.Consistent with previous studies, we also found that metabolic markers (including CPR, fasting glucose, and the TyG index) were notably higher in patients with gallstones than patients without.^[29,30]^

Earlier research has looked at the connection between one measure (CRP or TyG index) and gallstones. For example, researchers found a link between the severity of acute cholecystitis and CRP levels in a US population, while another cross-sectional study of a US population found that a 1-unit increase in the TyG index was associated with a 25% higher risk of gallstones. Unlike previous single indexes, which could only reflect inflammation or insulin resistance to a limited extent, our study’s innovative CTI index integrates the CRP and TyG indexes. CTI is a rapid, accessible, and cost-effective biochemical test that provides information on inflammation and insulin resistance status, as well as their reciprocal and synergistic associations with gallstones. In our study, a positive correlation was found between higher CTI and gallstone risk, regardless of whether CTI was used as a continuous or subgroup variable. This positive correlation was consistently present in different subgroups, indicating the stability of our results.Acute-phase inflammatory proteins are synthesized by the liver, and previous studies have shown that CRP is one such protein, and elevated levels of CRP can reflect systemic inflammation. Elevated CRP levels are often found in patients with gallstones, chronic cholecystitis or biliary tract infections.^[31]^ CRP is also closely related to lipid metabolism. High levels of CRP can inhibit the expression of apolipoprotein B, the main component of low-density lipoprotein, and may promote cholesterol crystallization by affecting its solubility in bile.^[32,33]^ Elevated CRP levels caused by chronic inflammation may also inhibit gallbladder smooth muscle contraction, leading to delayed bile excretion. Insulin resistance activates the sterol regulatory binding protein pathway, increasing hepatic cholesterol synthesis and leading to elevated cholesterol concentrations in bile.This results in supersaturated bile, the primary cause of cholesterol stone formation.^[34]^ Furthermore, insulin resistance inhibits the activity of cholesterol 7α-hydroxylase (CYP7A1), thereby reducing bile acid synthesis and decreasing bile’s ability to dissolve cholester.^[35]^ Animal studies have also shown that a decrease in the proportion of chenodeoxycholic acid in bile, along with an increase in hydrophobic bile acids, can further exacerbate cholesterol precipitation.^[36]^ An elevated TyG index is associated with systemic low-grade inflammation (e.g., elevated CRP and IL-6). Inflammatory factors can damage the gallbladder mucosa, reduce cholecystokinin sensitivity and lead to bile stasis.^[37]^ Patients with insulin resistance have a reduced gallbladder evacuation rate and prolonged bile retention time, which promotes crystal aggregation. The development of gallstones may be associated with chronic inflammation and insulin resistance. The synergistic effect of systemic inflammation and metabolic dysregulation creates an environment conducive to gallstone formation. Elevated levels of CRP activate nuclear factor-κB, which increases mucin (5AC) and immunoglobulin production, thus promoting cholesterol crystal formation and growth in the bile.^[33,38]^ Meanwhile, an elevated TyG promotes biliary stasis through impaired cholecystokinin signaling and autonomic neuropathy, leading to diminished gallbladder contractility.^[38]^ Hypertriglyceridemia, a core component of TyG, it exacerbates biliary cholesterol supersaturation by stimulating the overproduction of hepatic cholesterol while inhibiting the synthesis of bile acids via the downregulation of CYP7A by peroxisome proliferator-activated receptor alpha.^[39,40]^ Insulin resistance exacerbates oxidative stress further by decreasing glutathione peroxidase activity. This leads to the dysfunction of ABCG5/G8 transporter proteins and impaired biliary cholesterol efflux.^[2,41]^ CRP and TyG may interact in a bidirectional manner: pro-inflammatory cytokines are produced by adipose tissue. Examples include IL-6 and TNF-α, exacerbate insulin resistance; meanwhile, hyperglycemia promotes glucosylation.^[42]^ Hyperglycemia promotes the formation of glycation end-products, maintaining the inflammatory response. Multiple mechanisms integrate metabolic and inflammatory disturbances that contribute to the development of gallstones. Research into the exact processes linking CTI and gallstones has not yet reached a definitive conclusion. Proposed future research includes longitudinal clinical trials and detailed animal research.

The fact that CTI remains stable across different population groups (gender, age, and socioeconomic status) makes it a potentially more useful universal screening tool. In high-risk populations, CTI can inform targeted interventions. Statins, which lower CRP levels and improve lipid profiles simultaneously, may offer dual benefits in preventing gallstones.^[43]^ Similarly, GLP-1 agonists have been shown to enhance gallbladder evacuation in diabetic patients and may mitigate the risk associated with CTI.^[44]^

Our study boasts several key strengths. Firstly, by using nationally representative datasets, we discovered a correlation between CTI and gallstone disease. This provides new insights into the potential clinical relevance of this easily accessible metric. Secondly, CTI is a composite metric that provides a comprehensive overview of the inflammation and insulin resistance associated with gallstones, thus strengthening the robustness of our findings. Thirdly, we explored the consistency of the association between CTI and gallstone disease in different populations using a subgroup analysis. However, there are certain limitations to our study that must be acknowledged. Firstly, the cross-sectional design prevented us from clarifying the causal relationship between CTI and gallstones, which will require confirmation through longitudinal studies in the future. Secondly, despite adjusting for multiple covariates, it was not possible to fully mitigate the potential impact of all confounding factors. Thirdly, gallstone diagnosis relies heavily on self-reporting, which is subject to recall bias.

## 5. Conclusion

Our study, which used data from a US cross-sectional study, identified a significant correlation between CTI and gallstones. CTI demonstrated predictive ability in terms of gallstone risk stratification. However, these results require validation through prospective longitudinal studies.

## Acknowledgments

We would like to thank all participants in this study.

## Author contributions

**Conceptualization:** Qiu Su.

**Formal analysis:** Qiu Su.

**Methodology:** Qiu Su, Saimei Yang.

**Visualization:** Saimei Yang.

**Writing – review & editing:** Qiu Su, Qiyi Liu.
